# Alveolar-Capillary Membrane-Related Pulmonary Cells as a Target in Endotoxin-Induced Acute Lung Injury

**DOI:** 10.3390/ijms20040831

**Published:** 2019-02-15

**Authors:** Zuzana Nova, Henrieta Skovierova, Andrea Calkovska

**Affiliations:** 1 Department of Physiology and Biomedical Center Martin, Jessenius Faculty of Medicine in Martin, Comenius University in Bratislava, 03601 Martin, Slovakia; zuzanka.nova@gmail.com (Z.N.); calkovska@jfmed.uniba.sk (A.C.); 2 Biomedical Center Martin, Division of Molecular Medicine, Jessenius Faculty of Medicine in Martin, Comenius University in Bratislava, 03601 Martin, Slovakia

**Keywords:** acute respiratory distress syndrome, alveolar epithelial cells, endothelial cells, alveolar macrophages, fibroblasts, bacterial lipopolysaccharide

## Abstract

The main function of the lungs is oxygen transport from the atmosphere into the blood circulation, while it is necessary to keep the pulmonary tissue relatively free of pathogens. This is a difficult task because the respiratory system is constantly exposed to harmful substances entering the lungs by inhalation or via the blood stream. Individual types of lung cells are equipped with the mechanisms that maintain pulmonary homeostasis. Because of the clinical significance of acute respiratory distress syndrome (ARDS) the article refers to the physiological role of alveolar epithelial cells type I and II, endothelial cells, alveolar macrophages, and fibroblasts. However, all these cells can be damaged by lipopolysaccharide (LPS) which can reach the airspaces as the major component of the outer membrane of Gram-negative bacteria, and lead to local and systemic inflammation and toxicity. We also highlight a negative effect of LPS on lung cells related to alveolar-capillary barrier and their response to LPS exposure. Additionally, we describe the molecular mechanism of LPS signal transduction pathway in lung cells.

## 1. Introduction

**Figure 1:**
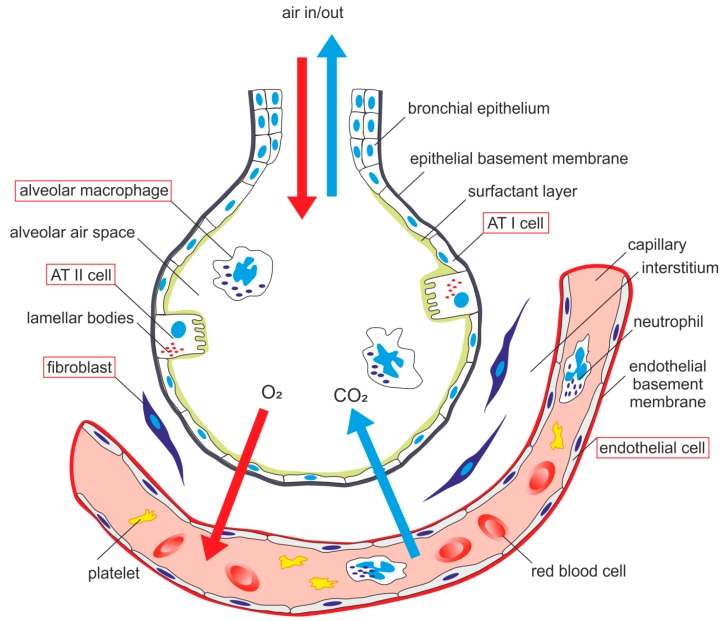
Schematic arrangement of alveolar-capillary membrane-related pulmonary cells. ATI cell—alveolar epithelial type I cell, ATII cell—alveolar epithelial type II cell.

From a histological point of view, the lung is a very complex organ. The pulmonary epithelium consists of two major cell types—alveolar type I (ATI) cells and alveolar type II (ATII) cells, also termed type I and type II pneumocytes. ATI together with ATII cells form a complete epithelial lining of the peripheral part of the lungs and play an important role in pulmonary homeostasis. The alveolar epithelium represents a mechanical barrier that protects lungs from environmental insults, it is actively involved in immune response of the lungs and contributes to the maintenance of alveolar surface fluid balance [1]. The alveolar epithelium is in close contact with the endothelial monolayer of the pulmonary capillary network. There are alveolar macrophages (AM) located close to the epithelial surface and capillary endothelial cells [2]. The interstitial space between these two kinds of cells contains fibroblasts [3] (Figure 1).

Lipopolysaccharide (LPS), also named as endotoxin, is a part of the outer membrane of Gram-negative bacteria. It consists of a hydrophilic polysaccharide (O-antigen), an oligosaccharide core and a highly toxic lipid A [4]. Based on morphology, bacteria can be divided into two groups, (i) smooth strains which express LPS with core oligosaccharide and O-antigen and (ii) rough strains expressing a complete or a truncated core oligosaccharide but lacking the O-antigen [5]. LPS has a pro-inflammatory effect and plays an important role in the pathogenesis of a Gram-negative bacterial infection. After entering the body, LPS stimulates the innate immunity and triggers biochemical and cellular responses that lead to the inflammation and toxicity [6]. Each cell type possesses common or cell-specific mechanisms by which it interacts with LPS when it enters the alveolus.

## 2. Mechanism of LPS Signal Transduction Pathway in Lung Cells

**Figure 2:**
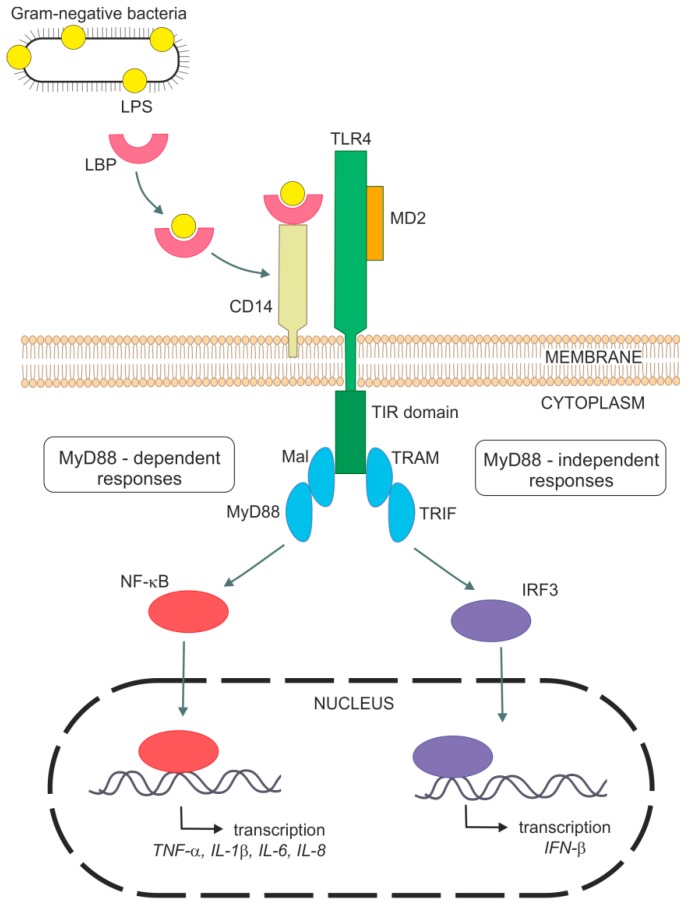
General mechanism of host immune response to Lipopolysaccharide (LPS) through TLR4 signaling. IFN-β—interferon β, IL—interleukin, IRF3—interferon regulatory factor 3, LBP—LPS binding molecule, LPS—lipopolysaccharide, Mal—MyD88 adaptor-like protein, MD2—myeloid differentiation protein 2, MyD88—myeloid differentiation factor 88, TIR domain—Toll/IL-1R homology domain, TLR4—toll-like receptor 4, TNF-α—tumor necrosis factor α, TRAM—TRIF-related adaptor molecule, TRIF—TIR-domain-containing adaptor protein.

As was mentioned above, LPS is a strong activator of the host innate immune system. Mechanisms of the innate immune response involves specific pattern-recognition receptors (PRR), which recognize conserved molecular structures of various pathogens so-called as pathogen-associated molecular patterns and trigger immunological responses. The most important members of PRRs are toll-like receptors (TLRs), which have ten different members in human. This integral membrane receptors consist of an extracellular domain responsible for a recognition of pathogen-associated molecular patterns (PAMP) and an intracellular signaling domain [7,8,9,10]. It has been shown that endotoxin-induced responses are mediated by TLR4 in cell cultures [11,12,13] and also in vivo [14,15,16]. For example, TLR4-deficient or spontaneous TLR4 mutants (C3H/HeJ and C57/10ScCr) were not able to respond to LPS and suppressed Gram-negative bacterial infection [14,15,17,18]. The requirement of TLR4 for LPS signaling is supported by genetic [19,20] and binding [21] data indicating a direct contact between endotoxin and TLR4. TLR4 stimulation by LPS is a complex process with a participation of several molecules. Figure 2 shows a general mechanism of LPS signaling. Specific features related to lung cells are mentioned within the next sections.

The first molecule implicated in the recognition of endotoxin is probably LPS-binding protein (LBP). This acute-phase plasma protein recognizes and binds to the lipid A part of LPS, extracts LPS from bacteria, solubilizes it and then transfers it to glycoprotein CD14 [22,23,24]. CD14 exists in two forms—membrane-bound CD14 (mCD14) in myeloid cells or soluble CD14 (sCD14) in blood, which allows it to respond to LPS in cells without mCD14, e.g., epithelial and endothelial cells [22,25,26,27]. CD14 reduces the amount of LPS required for cell responses on endotoxin exposure to picomolar concentrations [28,29]. Therefore both LBP and CD14, greatly enhance LPS binding to TLR4. Binding of CD14 to LPS was considered to be essential for the LPS signal transduction pathway. It was shown that CD14-deficient mice are to be resistant to endotoxin induced shock [28]. However, also CD14-independent binding of LPS probably exists [30,31,32]. CD14 is essential for TLR4 activation by the smooth form of LPS but not by the rough form [33,34]. TLR4 mediates cellular responses to endotoxin in cooperation with a small myeloid differentiation protein 2 (MD2) [35]. This protein seems to be essential for ligand recognition by TLR4. Requirement of MD2 for LPS responses was demonstrated by study using MD2 knockout mice, which did not react to LPS [36]. Similarly, the cell line with a point mutation in MD2 was unresponsive to LPS [37]. Extracellular domain of TLR4 forms a stable heterodimer with MD2. LPS binds to a hydrophobic pocket of MD2 and directly cause dimerization of the TLR4-MD2 complex. This triggers the recruitment of specific adaptor proteins to the intracellular domains and initiate a signaling cascade [38]. The intracellular TLR4 domains are structurally homologous to the interleukin-1 receptor (IL-1R) family and are called Toll/IL-1R homology (TIR) domains. Signaling adaptors also contains TIR domains, which interact with TIR domains of TLR4 after LPS binding [8]. TLR4 adaptors include myeloid differentiation factor 88 (MyD88), MyD88 adaptor-like protein (Mal), TIR-domain-containing adaptor protein (TRIF), and TRIF-related adaptor molecule (TRAM) [10,39].

Activated TLR4-MD2 complex can trigger two signal transduction pathways, (i) MyD88-dependent [40] and (ii) MyD88-independent pathway [41,42]. MyD88-dependent response requires Mal for efficient signaling and leads to rapid activation of NF-κB. MyD88-independent signaling is mediated through TRIF and TRAM, leading to phosphorylation and dimerization of interferon regulatory factor 3 (IRF3) resulting in release of interferon β (IFN-β). MyD88-independent pathway is also able to activate NF-κB signaling, but at late times. NF-κB activation leads to the production of various pro-inflammatory cytokines, like TNF-α, IL-1β, IL-6, and chemokines such as monocyte chemoattractant protein 1 (MCP-1), macrophage inflammatory protein 3α (MIP-3α), and IL-8 resulting in elimination of bacterial pathogens. However, overproduction of inflammatory molecules can cause septic shock [7,10,39,43,44,45].

TLR4-independent mechanisms of lung injury by LPS were also suggested. Hagar et al. [46] have reported that LPS-activated caspase-11 causes an endotoxic shock in mice lacking TLR4. Another group studied the effect of accumulation mode particles in the presence of LPS on airway inflammation. The inflammatory effect of inhaled particles was strongly influenced by LPS. Lung responses were enhanced in the case of TLR4 signalization, but inflammation was also detected in the absence of TLR4, suggesting other TLR4-dependent responses to LPS [47]. Furthermore, LPS can bind to the P2X7 receptor [48], which could have a role in LPS-induced ALI [49]. P2X7 is a transmembrane ion receptor widely expressed by immune cells [50]. Its activation leads to an increased membrane permeability for Ca^2+^ cations and changes in cellular ion homeostasis associated with a release of pro-inflammatory molecules [51,52,53]. Intratracheally administered LPS led to necrosis of alveolar macrophages and induction of ALI through P2X7 receptor pathway [52], which further supports the concept of TLR4-independent mechanisms of LPS action.

## 3. Alveolar Epithelial Type I Cells

ATI cells cover approximately 90% of the alveolar surface representing about 8% of the lung cell population. ATI cells are large flat cells with a surface area approximately 5000 μm^2^ and with a small number of organelles [54,55,56]. Very thin cytoplasm and a limited number of mitochondria cause extreme sensitivity of these cells to injury [57] and contribute to their vulnerability [58]. ATI cells were originally described as terminal differentiated cells without any ability to divide and change their phenotype [59]. However, very recently it was shown that ATI cells could change their phenotype to ATII cells and proliferate, for example after a partial pneumonectomy [60].

ATI cells are highly specialized for the key function of the lungs—the gas exchange between alveoli and capillary blood. Moreover, they are involved in regulation of cell proliferation, ion and water transport, peptide metabolism, macrophage function modulation and signaling pathways in peripheral lungs [57]. ATI cells have all pumps and ion channels for transcellular sodium transport [61]. Water permeability is mediated by aquaporins [62]. Participation of ATI cells in the innate immune response in the lungs is also important [63]. Besides, ATI cells are involved in pro-inflammatory response. There is a large contact surface area with alveolar macrophages. ATI cells are capable to express toll-like receptors (TLRs) and produce a number of pro-inflammatory cytokines in response to LPS stimulation and during pneumococcal infection [64].

### Interaction of ATI Cells with LPS

ATI cells can play an important role in the pathogenesis of lung damage. LPS at concentrations of 1, 5, 10, 15, and 20 μg/mL did not affect the viability of ATI cells in vitro at various time conditions [65]. However, it was shown that LPS can stimulate production of a wide range of inflammatory mediators [63]. LPS-induced inflammatory damage in the lungs is mediated by the receptor for advanced glycation end products (RAGE), which are expressed at high basal levels in ATI cells [66]. Its soluble isoform (sRAGE) was proposed as a marker of ATI cell damage [67]. LPS increases RAGE expression in ATI cells in dose-dependent manner. RAGE activates NF-κB, which further supports the transcription of many inflammatory factors, such as TNF-α or IL-1β [66]. It has been shown that primary rat ATI cells express TLR4 [68] but the interaction between TLR4 and LPS was more extensively studied with ATII cells (see Section 4.1).

## 4. Alveolar Epithelial Type II Cells

ATII cells form only 7% of the alveolar epithelium, representing 16% of all lung parenchymal cells [56]. ATII cells are small cuboid cells with surface area approximately 250 μm^2^ and a characteristic morphology with many lamellar bodies and apical microvilli [54].

The main function of ATII cells is synthesis, secretion, and recyclation of the pulmonary surfactant, which is required for maintaining sufficient respiratory surface area of the mammalian lungs at the end of expiration [55]. Surfactant also has an important function in the local defense mechanisms of the respiratory system. From the immunological point of view, surfactant proteins (SPs) SP-A and SP-D, which act as opsonins and regulate the function of inflammatory cells, are best characterized [69,70,71]. However, there is an increasing evidence that also SP-B and SP-C can be involved in the immunomodulation necessary for the respiratory system protection [72]. Furthermore, ATII cells produce cytokines and growth factors that could affect immune cells [73]. ATII cells also represent a major source of endogenous antimicrobial peptides (neutrophil α-defensins, β-defensins, katelicidin hCAP18/LL-37) in the lungs [74]. Therefore these cells can be referred to as “defenders of the alveolus”. Moreover, they are involved in sodium transport through sodium channels and thus help to keep the alveolar space relatively free of fluid [54].

ATII cells are considered to be a multipotent cell with high plasticity and capability of self-regeneration and trans-differentiation into ATI cells. They are responsible for repairing the damaged tissue. Furthermore, depletion of this cell population may lead to various pulmonary diseases [54]. For example, damage of ATII cells plays a key role in the pathogenesis of idiopathic pulmonary fibrosis (IPF). Administration of ATII cells directly to alveoli is thought to restore epithelial regeneration capability and thereby attenuate or even reverse the progression of IPF [75].

Epithelial cells may be damaged by various endogenous and exogenous factors. The most important exogenous factors are oxygen therapy [76] and artificial lung ventilation [77]. Pulmonary hypoxia is a possible consequence of many pathological conditions, including chronic obstructive pulmonary disease (COPD), lung tumors, pulmonary hypertension, edema and others [78].

### 4.1. Interaction of ATII Cells with LPS

Potential mechanisms of ATII cell response to microbial infection have been studied in various models, including primary cell cultures and continuous cell lines such as human lung carcinoma A549 cells. Because of the many difficulties with the isolation and maintenance of primary ATII cells in tissue cultures, associated with the loss of their morphological and biochemical characteristics [56,79]. A549 cells are extensively used as a model of ATII cells. However, the suitability of the A549 cell line as ATII model seems to be conflicting and is not fully investigated [79,80,81]. Regarding to surfactant production, data in the literature differ. A549 cells did not show any expression of surfactant proteins in the study of Mao et al. [82]. In contrary, others have reported expression of surfactant proteins in this cell line [83,84]. Moreover, Cooper et al. [79] confirmed multilamellar body development in A549 long term culture. It may serve as evidence of lung surfactant in A549 cells as presence of lamellar bodies, also in other cell types, indicates surfactant production [85]. Thus more extensive research of the suitability of A549 cells as ATII cellular model is needed.

Human ATII cells [86] and A549 cells [87] constitutively express TLR4. The exposure to LPS significantly increase expression of TLR4 in human ATII cells [88]. TLR4 of ATII cells seems to be located predominantly intracellularly [87,89]. TLR4 endocytosis induced by LPS is controlled by CD14 [90] which is expressed on the surface of ATII cells [89]. Alveolar epithelial cells were not shown to express mCD14 [87,91] but there is evidence that LPS induces release of cytokines in the presence of sCD14. It suggests a requirement of serum components for activation of these cells by LPS [92]. An important crosstalk between epithelial cells and neutrophils was reported [93]. Neutrophils were suggested as a donor of sCD14 for A549 during stimulation by endotoxin.

Exposure of alveolar epithelial cells to LPS modulates the surfactant proteins. The expression of SP-A was higher in A549 cells treated with LPS compared to the control. However, the expression of SP-D was not affected by LPS [93]. Similarly, the SP-A gene expression was increased in mice lung cells after LPS inhalation [94]. On the contrary, the expression of SP-B was decreased in ATII cells exposed to LPS [95], consistently with studies using an animal model of LPS-induced acute lung injury (ALI) [96]. It was assumed that the protein level could be reduced after stimulation with LPS. Moreover, LPS affects the interaction between SP-B and lipids and thus reduce the expression and membrane function of SP-B [95]. In the same way, SP-C expression was abnormally lower in ATII cells of LPS-exposed rats [97]. ATII cells express functional receptors for LPS, such as TLR2 and TLR4 [86]. TLR-dependent pathway could possibly explain LPS-caused lung inflammation and subsequent alterations in SPs expression in ATII cells. In our experiments, at least 1.5-fold decrease was present in the expression level of all SPs in the lungs of rats with intratracheal LPS at the dose 500 μg/kg, while the administration of LPS at the dose 1000 μg/kg even further potentiated this effect [98].

Endotoxin specifically activates TLRs, resulting in activation of the NF-κB signal transduction pathway and secretion of pro-inflammatory cytokines and chemokines like IL-6, IL-8, and type 1 interferons [83,93]. The effect of LPS on both types of cells, ATII and A549, leads to the reduction in cell viability and apoptosis induction, typical by the apoptotic bodies’ formation, DNA fragmentation, and chromatin condensation in time and concentration dependent manners [97,99]. Similarly, significantly enhanced apoptosis was present in ATII cells from animal models of LPS-induced ALI [100]. It has been shown that intratracheal administration of LPS in high doses directly caused the death of mice bronchial epithelial cells [101]. Excessive activity of ATII cells is another important cause of ALI. This kind of programmed cell death is characterized by the formation of autophagosomes, degradation of cytoplasmic content, and chromatin condensation [102]. Very recently, different time courses of autophagy and apoptosis in rat lung tissue during LPS-induced ALI were reported [103]. At early stages (1 and 2 h) of ALI, pulmonary cell death began with autophagy and peaked at 2 h. As ALI progressed, the level of apoptosis gradually increased and reached its maximum at later stages (6 h), while autophagy was decreased in a time-dependent manner. This suggests distinct roles of these two kinds of lung cell death at different stages of LPS-induced ALI. One of the main factors contributing to LPS-triggered death of alveolar epithelial cells may be an augmentation of intracellular reactive oxygen (ROS) and nitrogen species (RNS) including increased levels of cellular nitric oxide (NO). ROS and RNS are important apoptotic factors which may cause oxidative stress and subsequent cell apoptosis [99]. NO has been implicated in the pathogenesis of acute respiratory distress syndrome (ARDS) in animals and humans [104]. Previous studies described overproduction of NO, subsequent cellular oxidative stress and eventually cell death in different cell types exposed to LPS [105,106]. Considerably increased levels of intracellular ROS and NO were present also in A549 cells after LPS treatment [99]. Thus, LPS is able to directly damage alveolar epithelial cells through ROS-induced apoptotic mechanism.

### 4.2. Interaction of Pulmonary Surfactant with LPS

Interaction of pulmonary surfactant with LPS was recently reviewed by Kolomaznik et al. [6]. Once LPS enters the lungs, it also interacts with pulmonary surfactant. It has been shown that both Re-LPS [107] and S-LPS [108] interact with surfactant lipid films, resulting in fluidization of these films and alteration of its surface properties and subsequent surfactant dysfunction. Very recently, the effect of endotoxin on clinically used modified porcine surfactant poractant alpha was analyzed. Results from in vitro experiments have shown that 1% LPS caused structural disarrangement of surfactant molecule, including disruption of lamellar structure, leading to inability to reach low surface tension [98].

Besides surfactant lipid membranes, LPS directly interacts with SPs. Lung collectins (SP-A and SP-D), which significantly contribute to the surfactant homeostasis [109], interact with LPS through a signal regulatory protein alpha (SIRPα) receptor, which is typical for collectins or calreticulin/CD91 complex [110,111]. SP-A and SP-D interact with LPS in a different way. SP-A was shown to bind with Re-LPS to lipid A moiety dependently on calcium [112,113] and SP-D binds especially with S-LPS to an oligosaccharide region of LPS molecule through a carbohydrate recognition domain (CRD) [114]. The interaction of collectins with membrane LPS destabilizes the bacterial membrane and increases its permeability, which could explain their antimicrobial roles [115,116]. This is supported by the recent study in which the interaction of SP-A with LPS caused changes in conformation of LPS aggregates and reduced its biological activity [117]. It has been shown that lung collectins bind also with the CD14 molecule [113,118], TLR2 [119,120], TLR4 [120,121,122] and MD2 protein [119,122], which are involved in some responses to endotoxin. This suggests SP-A and SP-D are able to modulate cellular responses to endotoxin. The interaction of SP-A with CD14 was shown to inhibit binding of CD14 with S-LPS and thus may partially suppress inflammatory reaction. On the other hand, CD14 binding to Re-LPS seems to be more efficient in complex with SP-A. The binding of SP-D to CD14 inhibits interaction with both types of LPS [113].

The main function of small hydrophobic proteins SP-B and SP-C is to provide rapid adsorption of phospholipids to the air/liquid interface [123] and are required for biophysical activity of surfactant [124]. However, these two proteins could have also immunomodulatory properties. SP-C was found to be able to bind to the lipid A part of LPS through N-terminal domain [125] and could modulate activity of alveolar macrophages [126,127] (see Section 6). Similarly to SP-A, SP-C binds with CD14 [111] and subsequent modulation of CD14 conformation allows to bind LPS to CD14 more efficiently [128].

## 5. Endothelial Cells

Endothelial cells (cell surface area approximately 1350 μm^2^) form a thin monolayer lining the inner surface of the blood vessels and create an interface between the blood and tissues [3,129]. Endothelium is not only a passive barrier, but it also actively regulates vessel permeability, passage of blood components into surrounding tissues, vascular tone, signaling, and angiogenesis [130]. Endothelial cells are capable of attracting a wide range of immune cells and they are thought to play a role in antigen presentation [131].

Normal function of the endothelial barrier may be impaired by various external influences, including inflammation, sepsis, tumor growth, diabetes, atherosclerosis and other diseases. Inflammatory factors (e.g., histamine, bradykinin or platelet activating factor) increase capillary permeability and allow substances including plasma proteins to enter alveoli. It is an important step in pathogenesis of ARDS [132].

### Interaction of Endothelial Cells with LPS

Dysfunction of alveolar-capillary barrier is a characteristic feature of lung injury. This barrier is susceptible to bacterial infection that initiates a number of pathological events in the cells and finally leads to barrier breakdown [133]. An increased endothelial permeability is one of the major pathological characteristics of ALI [134]. Endothelium does not express mCD14. The interaction of LPS with endothelial cells is mediated through a sCD14 present in blood and TLR4 receptor which is expressed by these cells [135]. If sCD14 is not present, endothelial cells are resistant to LPS-triggered apoptosis. Similarly, antibodies against CD14 inhibit LPS toxicity in endothelium [136]. LPS binds to the surface of endothelial cells during infection and activates endothelial signaling pathways and secretion of inflammatory mediators such as IL-1ß, IL-6, IL-8 and TNF-α [133,137]. These mediators induce ROS production, secretion of chemokines and adhesion molecules, reduction of anti-inflammatory mediators, and leukocyte transmigration [138]. Increased production of pro-inflammatory cytokines affects the microtubule arrangement in pulmonary endothelium [139]. There are interactions between circulating leukocytes and vascular endothelium. Monocytes adhesion to activated endothelium and their subsequent proliferation is a cause of atherosclerotic changes. Chemokines produced by endothelial cells facilitates the movement of circulating monocytes to atherosclerotic vessels and site of infection [138,140]. In addition to the increased levels of inflammatory cytokines, high expression of ICAM-1 and VCAM-1 molecules was observed in LPS-activated endothelial cells. These molecules are known to mediate endothelial cell immune response during bacterial infections [133,141]. LPS also strongly induces a key complement factors such as C1s, C1R, C3 [133] consistently with the current knowledge of their involvement in pulmonary host defense [142] and endothelial cell damage during acute lung injury and sepsis [143].

Pro-inflammatory cytokines secreted by endothelial cells exposed to LPS also lead to the immunological activation and subsequent dysfunction of the epithelial barrier. IL-6 is known to modulate barrier properties of epithelial cells [144]. Janga et al. [133] compared the concentration of cytokines in monocultures of epithelial or endothelial cells and from their co-culture after LPS exposure. Epithelial cells secreted much lower amounts of inflammatory cytokines. After exposure to soluble factors from LPS-treated endothelial cells, epithelial cells reacted by an up-regulation of SP-A and significant down-regulation of SP-C and SP-D gene expression. These changes could be due to more the result of paracrine factors, for example inflammatory cytokines secreted by endothelial cells than the direct effect of LPS on epithelial cells.

## 6. Alveolar Macrophages

Alveolar macrophages (AM) are the major resident cell line in the bronchoalveolar space. Together with alveolar epithelial cells, they provide the immediate response to toxic substances and pathogens that reach the lower respiratory tract [145]. Another important role of AM is degradation of “used” surfactant, by which they prevent intra-alveolar accumulation of lipids and proteins and thus help to maintain homeostasis [146]. AM are large mononuclear phagocytic cells. Their precursors, monocytes, are formed from the hematopoietic stem cells in the bone marrow and they are released into the bloodstream. Monocytes differentiate into macrophages and dendritic cells during migration into tissues [147]. Three basic populations of macrophages with different functions were described—M1, M2, and regulatory macrophages. Classically activated M1 macrophages, which are produced under the influence of interferon IFN-γ, are generally characterized by pro-inflammatory properties. They mediate host defense against various bacteria or viruses and play a role in antitumor immunity. Alternatively activated M2 macrophages, induced by IL-4 and IL-3 cytokines have anti-inflammatory functions and regulate wound healing. Regulatory macrophages secrete large amounts of IL-10, an essential anti-inflammatory cytokine, which is responsible to control the excessive inflammatory and autoimmune reactions [148,149].

AM in healthy individuals cannot be strictly classified into the M1 and M2 populations. The final phenotype depends on the interaction of cooperating pro- and anti-inflammatory factors. Overgrowth of M2 macrophages is a sign of many inflammatory lung diseases in humans and rodents [149] and studies suggest an important role of this population in the pathogenesis of pulmonary fibrosis [150,151]. Although M2 macrophages participate in the repair of damaged lungs, persistence of this phenotype contributes to the excessive formation of pro-fibrotic factors associated with airway remodeling typical for chronic asthma [152]. 

Recent research has highlighted the complex communication between AM and airway epithelium [153,154], which leads to the release of a wide range of cytokines and chemokines and is a key modulator of inflammatory responses [149]. Epithelium maintains macrophages in limited anti-inflammatory phenotype via interaction with the membrane glycoprotein CD200, IL-10 and transforming growth factor β (TGF-β) [155]. Surfactant proteins SP-A and SP-D are involved in negative regulation, too. In addition to binding to SIRPα, SP-A promotes the anti-inflammatory state in the lungs by inhibition of the C1 complex formation which is required for complement activation [156]. SP-A can also block binding of ligands to TLR2 and TLR4 receptors [122] and it is able to regulate TLRs expression in human macrophages [157]. In another studies SP-A decreased release of TNF-α from LPS-stimulated rat and human AM [110,158]. SP-D has a key regulatory role, because SP-D deficient mice exhibit constitutive activation of AM [159]. However, in contrast to SP-A, SP-D was shown to slightly enhance TNF-α production in LPS-stimulated AM [160]. Another anti-inflammatory mechanism of the lung may be represented by SP-C, which is able to bind with LPS. SP-C associated with DPPC vesicles was shown to inhibit LPS binding to RAW254.7 macrophages and endotoxin-induced TNF-α production by peritoneal and alveolar macrophages [126]. Glasser et al. [127] suggested that SP-C deficiency disrupts the activation of AM and leads to the decrease in phagocytosis and pathogen clearance. Destruction of the airway epithelium and loss of its regulatory ligands can lead to pro-inflammatory response of AM to airway antigens. For example, the absence of CD200 results in a doubling of the number of AM and spontaneous regulation of activation markers [161]. An important determinant of pro- or anti-inflammatory response of macrophages are apoptotic and necrotic cells. In the first case, AM begins to act anti-inflammatory to prevent inflammatory reactions against their own proteins. Apoptotic cells also exhibit increased expression of CD200. Conversely, necrosis and secondary necrosis, which occurs when apoptotic cells are not removed, release cellular components associated with pro-inflammatory damage [149].

### Interaction of Alveolar Macrophages with LPS

LPS belongs to the classic activator of macrophages [148]. Response of AM to LPS and subsequent release of cytokines is dependent on TLR4 [162]. AM constitutively express TLR4 only at low levels but TLR4 expression is quickly enhanced after LPS exposure [88]. LPS binds to TLR4 receptor and leads to activation of NF-κB, production of various inflammatory mediators (TNF-α, IL-6), and ultimately cell damage and clinical manifestations of sepsis [163,164]. An increased expression of 10 different mRNAs encoding pro-inflammatory cytokines and chemokines has been reported in LPS-activated AM [165]. Furthermore, intratracheally administered LPS induced the release of pro-interleukin-1α (IL-1α) from necrotic AM which subsequently activated endothelial cells and increased the vascular permeability [52].

The ability of macrophages to phagocyte bacteria is significantly increased after exposure to a low concentration of LPS (0.1 μg/mL LPS). A slight increase was also observed at higher concentrations of LPS [166]. The production of cytokines by LPS-stimulated immune cells is time- and dose-dependent, whereas TNF-α can be released from macrophages one hour after LPS stimulation [167]. Another study demonstrated that LPS-mediated induction of TNF-α inhibits the phagocytic ability of macrophages [168]. Thus, insufficient elevation of phagocytosis after stimulation with higher LPS concentration can be attributed to LPS-mediated early induction of TNF-α [166].

## 7. Lung Fibroblasts

Lung fibroblasts play a key role in all stages of lung development, including the development of alveolar units [169]. A number of lung fibroblast subunits have been defined, including lipo-fibroblasts, myofibroblasts and their precursors [170]. Under physiological conditions, fibroblasts produce only a small amount of extracellular matrix. After tissue damage, fibroblasts differentiate into contractile and secretory myofibroblasts, migrate to the site of injury, synthesize extracellular matrix components and contribute to tissue repair and wound healing. However, when these processes become excessive, they can cause a severe organ damage [171].

### Interaction of Lung Fibroblasts with LPS

Only a few studies have been focused on LPS effects on fibroblasts. There is an evidence that lung fibroblasts express TLR4 [172]. LPS can directly induce proliferation of lung fibroblasts through TLR4 receptors and thereby accelerate pulmonary fibrosis in the early stages of ALI [173]. Aberrant proliferation of pulmonary fibroblasts was detected in bleomycin-induced pulmonary fibrosis [174], IPF patients [175] and LPS induced ALI [176]. Furthermore, LPS directly induces collagen secretion in primary cultured murine pulmonary fibroblasts via TLR4 signaling [172]. Another important factor in IPF pathogenesis is the communication between fibroblasts and alveolar epithelium. Mediators derived from alveolar epithelial cells control migration, proliferation, activation and differentiation of fibroblasts, leading to accumulation of myofibroblasts and extracellular matrix in the lungs. Myofibroblasts in turn secrete mediators that amplify epithelial cell damage and apoptosis, creating a vicious circle of pro-fibrotic interactions [177].

## 8. Conclusions

The effect of LPS on the pulmonary alveoli is complex. Endotoxin causes lung damage by interacting with individual types of lung cells and initiating the secretion of various inflammatory mediators. This process results in disruption of alveolar epithelial and endothelial barriers. LPS also interferes with the ability of ATII cells to produce pulmonary surfactant and LPS itself binds to surfactant proteins and phospholipids leading to surfactant inactivation. The leak of protein-reach plasma into the airspaces and surfactant inactivation is an initial step of ARDS and thus LPS-induced lung injury may be a serious clinical problem. Except the standard measures included in ARDS treatment protocol, novel approaches such as administration of mesenchymal stem cells, which could possibly resolve pulmonary tissue damage, has to be considered.

## Abbreviations

ALI	acute lung injury
AM	alveolar macrophages
ARDS	acute respiratory distress syndrome
ATI cells	alveolar epithelial type I cells
ATII cells	alveolar epithelial type II cells
COPD	chronic obstructive pulmonary disease
DPPC	dipalmitoylphosphatidylcholine
ICAM-1	intercellular Adhesion Molecule 1
IFN- β	interferon β
IFN- γ	interferon γ
IL	interleukin
IL-1R	interleukin-1 receptor
IPF	idiopathic pulmonary fibrosis
LBP	LPS-binding protein
Mal	MyD88 adaptor-like protein
mCD14	membrane-bound CD14
MCP-1	monocyte chemoattractant protein 1
MD2	myeloid differentiation protein 2
MIP-3α	macrophage inflammatory protein 3α
MyD88	myeloid differentiation factor 88
NF- κB	nuclear factor-κB
NO	nitric oxide
PAMP	pathogen-associated molecular pattern
PPRs	pattern-recognition receptors
RAGE	receptor for advanced glycation end products
RNS	reactive nitrogen species
ROS	reactive oxygen species
sCD14	Soluble CD14
SIRPα	Signal regulatory protein α
SP-A	surfactant protein A
SP-B	surfactant protein B
SP-C	surfactant protein C
SP-D	surfactant protein D
SPs	surfactant proteins
sRAGE	soluble isoform of receptor for advanced glycation end products
TGF-β	transforming growth factor β
TIR domains	Toll/IL-1R homology domains
TLRs	toll-like receptors
TNF- α	tumor necrosis factor α
TRAM	TRIF-related adaptor molecule
TRIF	TIR-domain-containing adaptor protein
VCAM-1	vascular cell adhesion molecule 1

## References

[1] Guillot L., Nathan N., Tabary O., Thouvenin G., Le Rouzic P., Corvol H., Amselem S., Clement A. (2013). Alveolar Epithelial Cells: Master Regulators of Lung Homeostasis. Int. J. Biochem. Cell Biol..

[2] Losa D., Chanson M. (2015). The Lung Communication Network. Cell. Mol. Life Sci..

[3] Weibel E.R. (2017). Lung Morphometry: The Link between Structure and Function. Cell Tissue Res..

[4] Nikaido H. (2003). Molecular basis of bacterial outer membrane permeability revisited. Microbiol. Mol. Biol. Rev..

[5] Zhang G., Meredith T.C., Kahne D. (2013). On the essentiality of lipopolysaccharide to Gram-negative bacteria. Curr. Opin. Microbiol..

[6] Kolomaznik M., Nova Z., Calkovska A. (2017). Pulmonary Surfactant and Bacterial Lipopolysaccharide: The Interaction and its Functional Consequences. Physiol. Res..

[7] Akira S., Takeda K. (2004). Toll-like receptor signalling. Nat. Rev. Immunol..

[8] Akira S., Uematsu S., Takeuchi O. (2006). Pathogen recognition and innate immunity. Cell.

[9] Beutler B.A. (2009). TLRs and innate immunity. Blood.

[10] Płóciennikowska A., Hromada-Judycka A., Borzęcka K., Kwiatkowska K. (2015). Co-operation of TLR4 and raft proteins in LPS-induced pro-inflammatory signaling. Cell. Mol. Life Sci..

[11] Chow J.C., Young D.W., Golenbock D.T., Christ W.J., Gusovsky F. (1999). Toll-like receptor-4 mediates lipopolysaccharide-induced signal transduction. J. Biol. Chem..

[12] Yang H., Young D.W., Gusovsky F., Chow J.C. (2000). Cellular events mediated by lipopolysaccharide-stimulated toll-like receptor 4. MD-2 is required for activation of mitogen-activated protein kinases and Elk-1. J. Biol. Chem..

[13] Nativel B., Couret D., Giraud P., Meilhac O., d’Hellencourt C.L., Viranaïcken W., Da Silva C.R. (2017). Porphyromonas gingivalis lipopolysaccharides act exclusively through TLR4 with a resilience between mouse and human. Sci. Rep..

[14] Hoshino K., Takeuchi O., Kawai T., Sanjo H., Ogawa T., Takeda Y., Takeda K., Akira S. (1999). Cutting edge: Toll-like receptor 4 (TLR4)-deficient mice are hyporesponsive to lipopolysaccharide: Evidence for TLR4 as the Lps gene product. J. Immunol..

[15] Takeuchi O., Hoshino K., Kawai T., Sanjo H., Takada H., Ogawa T., Takeda K., Akira S. (1999). Differential roles of TLR2 and TLR4 in recognition of gram-negative and gram-positive bacterial cell wall components. Immunity.

[16] Wang P., Han X., Mo B., Huang G., Wang C. (2017). LPS enhances TLR4 expression and IFN-γ production via the TLR4/IRAK/NF-κB signaling pathway in rat pulmonary arterial smooth muscle cells. Mol. Med. Rep..

[17] Poltorak A., He X., Smirnova I., Liu M.Y., van Huffel C., Du X., Birdwell D., Alejos E., Silva M., Galanos C. (1998). Defective LPS signaling in C3H/HeJ and C57BL/10ScCr mice: Mutations in Tlr4 gene. Science.

[18] Arbour N.C., Lorenz E., Schutte B.C., Zabner J., Kline J.N., Jones M., Frees K., Watt J.L., Schwartz D.A. (2000). TLR4 mutations are associated with endotoxin hyporesponsiveness in humans. Nat. Genet..

[19] Poltorak A., Ricciardi-Castagnoli P., Citterio S., Beutler B. (2000). Physical contact between LPS and Tlr4 revealed by genetic complementation. Proc. Natl. Acad. Sci. USA.

[20] Lien E., Means T.K., Heine H., Yoshimura A., Kusumoto S., Fukase K., Fenton M.J., Oikawa M., Qureshi N., Monks B. (2000). Toll-like receptor 4 imparts ligand-specific recognition of bacterial lipopolysaccharide. J. Clin Investig..

[21] Da Silva Correia J., Soldau K., Christen U., Tobias P.S., Ulevitch R.J. (2001). Lipopolysaccharide is in close proximity to each of the proteins in its membrane receptor complex: Transfer from CD14 to TLR4 and MD-2. J. Biol. Chem..

[22] Schumann R.R., Leong S.R., Flaggs G.W., Gray P.W., Wright S.D., Mathison J.C., Tobias P.S., Ulevitch R.J. (1990). Structure and function of lipopolysaccharide binding protein. Science.

[23] Fujihara M., Muroi M., Tanamoto K., Suzuki T., Azuma H., Ikeda H. (2003). Molecular mechanisms of macrophage activation and deactivation by lipopolysaccharide: Roles of the receptor complex. Pharmacol. Ther..

[24] Kim S.J., Kim H.M. (2017). Dynamic lipopolysaccharide transfer cascade to TLR4/MD2 complex via LBP and CD14. BMB Rep..

[25] Pugin J., Heumann I.D., Tomasz A., Kravchenko V.V., Akamatsu Y., Nishijima M., Glauser M.P., Tobias P.S., Ulevitch R.J. (1994). CD14 is a pattern recognition receptor. Immunity.

[26] Tapping R.I., Tobias P.S. (2000). Soluble CD14-mediated cellular responses to lipopolysaccharide. Chem. Immunol..

[27] Zanoni I., Granucci F. (2013). Role of CD14 in host protection against infections and in metabolism regulation. Front. Cell. Infect. Microbiol..

[28] Haziot A., Ferrero E., Köntgen F., Hijiya N., Yamamoto S., Silver J., Stewart C.L., Goyert S.M. (1996). Resistance to endotoxin shock and reduced dissemination of Gram-negative bacteria in CD14-deficient mice. Immunity.

[29] Gioannini T.L., Teghanemt A., Zhang D., Coussens N.P., Dockstader W., Ramaswamy S., Weiss J.P. (2004). Isolation of an endotoxin–MD 2 complex that produces Toll like receptor 4 dependent cell activation at picomolar concentrations. Proc. Natl. Acad. Sci. USA.

[30] Haziot A., Lin X.Y., Zhang F., Goyert S.M. (1998). The induction of acute phase proteins by lipopolysaccharide uses a novel pathway that is CD14-independent. J. Immunol..

[31] Kimura S., Tamamura T., Nakagawa I., Koga T., Fujiwara T., Hamada S. (2000). CD14-dependent and independent pathways in lipopolysaccharide-induced activation of a murine B-cell line, CH12. LX. Scand. J. Immunol..

[32] Watanabe S., Kumazawa Y., Inoue J. (2013). Liposomal lipopolysaccharide initiates TRIF-dependent signaling pathway independent of CD14. PLoS ONE.

[33] Jiang Z., Georgel P., Du X., Shamel L., Sovath S., Mudd S., Huber M., Kalis C., Keck S., Galanos C. (2005). CD14 is required for MyD88-independent LPS signaling. Nat. Immunol..

[34] Huber M., Kalis C., Keck S., Jiang Z., Georgel P., Du X., Shamel L., Sovath S., Mudd S., Beutler B. (2006). R-form LPS, the master key to the activation ofTLR4/MD-2-positive cells. Eur. J. Immunol..

[35] Beutler B., Rietschel E. (2003). Innate immune sensing and its roots: The story of endotoxin. Nat. Rev. Immunol..

[36] Nagai Y., Akashi S., Nagafuku M., Ogata M., Iwakura Y., Akira S., Kitamura T., Kosugi A., Kimoto M., Miyake K. (2002). Essential role of MD-2 in LPS responsiveness and TLR4 distribution. Nat. Immunol..

[37] Schromm A.B., Lien E., Henneke P., Chow J.C., Yoshimura A., Heine H., Latz E., Monks B.G., Schwartz D.A., Miyake K. (2001). Molecular genetic analysis of an endotoxin nonresponder mutant cell line: A point mutation in a conserved region of MD-2 abolishes endotoxin-induced signalling. J. Exp. Med..

[38] Park B.S., Song D.H., Kim H.M., Choi B.S., Lee H., Lee J.O. (2009). The structural basis of lipopolysaccharide recognition by the TLR4–MD-2 complex. Nature.

[39] O’Neill L.A., Bowie A.G. (2007). The family of five: TIR domain containing adaptors in Toll like receptor signalling. Nat. Rev. Immunol..

[40] Feng C.G., Scanga C.A., Collazo-Custodio C.M., Cheever A.W., Hieny S., Caspar P., Sher A. (2003). Mice lacking myeloid differentiation factor 88 display profound defects in host resistance and immune responses to Mycobacterium avium infection not exhibited by Toll-like receptor 2 (TLR2)- and TLR4-deficient animals. J. Immunol..

[41] Hoebe K., Du X., Georgel P., Janssen E., Tabeta K., Kim S.O., Goode J., Lin P., Mann N., Mudd S. (2003). Identification of Lps2 as a key transducer of MyD88-independent TIR signalling. Nature.

[42] Oshiumi H., Sasai M., Shida K., Fujita T., Matsumoto M., Seya T. (2003). TIR-containing adapter molecule (TICAM)-2, a bridging adapter recruiting to toll-like receptor 4 TICAM-1 that induces interferon-beta. J. Biol. Chem..

[43] Pålsson-McDermott E.M., O’Neill L.A. (2004). Signal transduction by the lipopolysaccharide receptor, Toll-like receptor-4. Immunology.

[44] Zughaier S.M., Zimmer S.M., Datta A., Carlson R.W., Stephens D.S. (2005). Differential Induction of the Toll-Like Receptor 4-MyD88-Dependent and -Independent Signaling Pathways by Endotoxins. Infect. Immun..

[45] Kieser K.J., Kagan J.C. (2017). Multi-receptor detection of individual bacterial products by the innate immune system. Nat. Rev. Immunol..

[46] Hagar J.A., Powell D.A., Aachoui Y., Ernst R.K., Miao E.A. (2013). Cytoplasmic LPS activates caspase-11: Implications in TLR4-independent endotoxic shock. Science.

[47] Fonceca A.M., Zosky G.R., Bozanich E.M., Sutanto E.N., Kicic A., McNamara P.S., Knight D.A., Sly P.D., Turner D.J., Stick S.M. (2018). Accumulation mode particles and LPS exposure induce TLR-4 dependent and independent inflammatory responses in the lung. Respir. Res..

[48] Denlinger L.C., Fisette P.L., Sommer J.A., Watters J.J., Prabhu U., Dubyak G.R., Proctor R.A., Bertics P.J. (2001). Cutting edge: The nucleotide receptor P2X7 contains multiple protein- and lipid-interaction motifs including a potential binding site for bacterial lipopolysaccharide. J. Immunol..

[49] Monção-Ribeiro L.C., Cagido V.R., Lima-Murad G., Santana P.T., Riva D.R., Borojevic R., Zin W.A., Cavalcante M.C., Riça I., Brando-Lima A.C. (2011). Lipopolysaccharide-induced lung injury: Role of P2X7 receptor. Respir. Physiol. Neurobiol..

[50] Volonté C., Apolloni S., Skaper S.D., Burnstock G. (2012). P2X7 receptors: Channels, pores and more. CNS Neurol. Disord. Drug Targets.

[51] Costa-Junior H.M., Sarmento Vieira F., Coutinho-Silva R. (2011). C terminus of the P2X7 receptor: Treasure hunting. Purinergic Signal..

[52] Dagvadorj J., Shimada K., Chen S., Jones H.D., Tumurkhuu G., Zhang W., Wawrowsky K.A., Crother T.R., Arditi M. (2015). Lipopolysaccharide Induces Alveolar Macrophage Necrosis via CD14 and the P2X7 Receptor Leading to Interleukin-1α Release. Immunity.

[53] Di Virgilio F., Dal Ben D., Sarti A.C., Giuliani A.L., Falzoni S. (2017). The P2X7 Receptor in Infection and Inflammation. Immunity.

[54] Mason R.J. (2006). Biology of Alveolar Type II Cells. Respirology.

[55] Lopez-Rodriguez E., Perez-Gil J. (2014). Structure-function relationships in pulmonary surfactant membranes: From biophysics to therapy. Biochim. Biophys. Acta.

[56] Beers M.F., Moodley Y. (2017). When Is an Alveolar Type 2 Cell an Alveolar Type 2 Cell? A Conundrum for Lung Stem Cell Biology and Regenerative Medicine. Am. J. Respir. Cell Mol. Biol..

[57] Herzog E.L., Brody A.R., Colby T.V., Mason R., Williams M.C. (2008). Knowns and unknowns of the alveolus. Proc. Am. Thorac. Soc..

[58] Griffiths M.J., Bonnet D., Janes S.M. (2005). Stem Cells of the Alveolar epithelium. Lancet.

[59] Williams M.C. (2003). Alveolar Type I Cells: Molecular Phenotype and Development. Annu. Rev. Physiol..

[60] Jain R., Barkauskas C.E., Takeda N., Bowie E.J., Aghajanian H., Wang Q., Padmanabhan A., Manderfield L.J., Gupta M., Li D. (2015). Plasticity of Hopx(+) Type I Alveolar Cells to Regenerate Type II Cells in the Lung. Nat. Commun..

[61] Johnson M.D., Widdicombe J.H., Allen L., Barbry P., Dobbs L.G. (2002). Alveolar epithelial type I cells contain transport proteins and transport sodium, supporting an active role for type I cells in regulation of lung liquid homeostasis. Proc. Natl. Acad. Sci. USA.

[62] Toczylowska-Maminska R., Dolowy K. (2012). Ion transporting proteins of human bronchial epithelium. J. Cell. Biochem..

[63] Wong M.H., Johnson M.D. (2013). Differential response of primary alveolar type I and type II cells to LPS stimulation. PLoS ONE.

[64] Yamamoto K., Ferrari J.D., Cao Y., Ramirez M.I., Jones M.R., Quinton L.J., Mizgerd J.P. (2012). Type I alveolar epithelial cells mount innate immune responses during pneumococcal pneumonia. J. Immunol..

[65] Nova Z., Mokra D., Vidomanova E., Kolomaznik M., Skovierova H., Halasova E., Calkovska A. (2017). Effect of lipopolysaccharide on alveolar epithelial type II cells. Acta Physiol..

[66] Li Y., Wu R., Zhao S., Cheng H., Ji P., Yu M., Tian Z. (2014). RAGE/NF-κB pathway mediates lipopolysaccharide-induced inflammation in alveolar type I epithelial cells isolated from neonate rats. Inflammation.

[67] Uchida T., Shirasawa M., Ware L.B., Kojima K., Hata Y., Makita K., Mednick G., Matthay Z.A., Matthay M.A. (2006). Receptor for advanced glycation end-products is a marker of type I cell injury in acute lung injury. Am. J. Respir. Crit. Care Med..

[68] Wong M.H., Chapin O.C., Johnson M.D. (2012). LPS-stimulated cytokine production in type I cells is modulated by the renin-angiotensin system. Am. J. Respir. Cell Mol. Biol..

[69] Wright J.R. (2005). Immunoregulatory functions of surfactant proteins. Nat. Rev. Immunol..

[70] Kuroki Y., Takahashi M., Nishitani C. (2007). Pulmonary collectins in innate imunity of the lung. Cell. Microbiol..

[71] Uhliarova B., Kopincova J., Adamkov M., Svec M., Calkovska A. (2016). Surfactant proteins A and D are related to severity of the disease, pathogenic bacteria and comorbidity in patients with chronic rhinosinusitis with and without nasal polyps. Clin. Otolaryngol..

[72] Mulugeta S., Beers M.F. (2006). Surfactant protein C: Its unique properties and emerging immunomodulatory role in the lung. Microbes Infect..

[73] Zissel G., Ernst M., Rabe K., Papadopoulos T., Magnussen H., Schlaak M., Muller-Quernheim J. (2000). Human alveolar epithelial cells type II are capable of regulating T-cell activity. J. Investig. Med..

[74] Hiemstra P.S., Amatngalim G.D., van der Does A.M., Taube C. (2016). Antimicrobial Peptides and Innate Lung Defenses: Role in Infectious and Noninfectious Lung Diseases and Therapeutic Applications. Chest.

[75] Serrano-Mollar A., Gay-Jordi G., Guillamat-Prats R., Closa D., Hernandez-Gonzalez F., Marin P., Burgos F., Martorell J., Sanchez M., Arguis P. (2016). Safety and Tolerability of Alveolar Type II Cell Transplantation in Idiopathic Pulmonary Fibrosis. Chest.

[76] Zaher T.E., Miller E.J., Morrow D.M., Javdan M., Mantell L.L. (2007). Hyperoxia-induced signal transduction pathways in pulmonary epithelial cells. Free Radic. Biol. Med..

[77] De Prost N., Dreyfuss D. (2012). How to prevent ventilator-induced lung injury?. Minerva Anestesiol..

[78] Shimoda L.A., Semenza G.L. (2011). HIF and the lung: Role of hypoxia-inducible factors in pulmonary development and disease. Am. J. Respir. Crit. Care Med..

[79] Cooper J.R., Abdullatif M.B., Burnett E.C., Kempsell K.E., Conforti F., Tolley H., Collins J.E., Davies D.E. (2016). Long Term Culture of the A549 Cancer Cell Line Promotes Multilamellar Body Formation and Differentiation towards an Alveolar Type II Pneumocyte Phenotype. PLoS ONE.

[80] Swain R.J., Kemp S.J., Goldstraw P., Tetley T.D., Stevens M.M. (2010). Assessment of Cell Line Models of Primary Human Cells by Raman Spectral Phenotyping. Biophys. J..

[81] Corbière V., Dirix V., Norrenberg S., Cappello M., Remmelink M., Mascart F. (2011). Phenotypic characteristics of human type II alveolar epithelial cells suitable for antigen presentation to T lymphocytes. Respir. Res..

[82] Mao P., Wu S., Li J., Fu W., He W., Liu X., Slutsky A.S., Zhang H., Li Y. (2015). Human alveolar epithelial type II cells in primary culture. Physiol. Rep..

[83] Chuang C.Y., Chen T.L., Chen R.M. (2009). Molecular mechanisms of lipopolysaccharide-caused induction of surfactant protein-A gene expression in human alveolar epithelial A549 cells. Toxicol. Lett..

[84] Rucka Z., Vanhara P., Koutna I., Tesarova L., Potesilova M., Stejskal S., Simara P., Dolezel J., Zvonicek V., Coufal O. (2013). Differential effects of insulin and dexamethasone on pulmonary surfactant-associated genes and proteins in A549 and H441 cells and lung tissue. Int. J. Mol. Med..

[85] Schmitz G., Müller G. (1991). Structure and function of lamellar bodies, lipid-protein complexes involved in storage and secretion of cellular lipids. J. Lipid Res..

[86] Armstrong L., Medford A.R., Uppington K.M., Robertson J., Witherden I.R., Tetley T.D., Millar A.B. (2004). Expression of functional toll-like receptor-2 and -4 on alveolar epithelial cells. Am. J. Respir. Cell Mol. Biol..

[87] Guillot L., Medjane S., Le-Barillec K., Balloy V., Danel C., Chignard M., Si-Tahar M. (2004). Response of human pulmonary epithelial cells to lipopolysaccharide involves Toll-like receptor 4 (TLR4)-dependent signaling pathways: Evidence for an intracellular compartmentalization of TLR4. J. Biol. Chem..

[88] Sender V., Stamme C. (2014). Lung cell-specific modulation of LPS-induced TLR4 receptor and adaptor localization. Commun. Integr. Biol..

[89] Thorley A.J., Grandolfo D., Lim E., Goldstraw P., Young A., Tetley T.D. (2011). Innate immune responses to bacterial ligands in the peripheral human lung—Role of alveolar epithelial TLR expression and signalling. PLoS ONE.

[90] Zanoni I., Ostuni R., Marek L.R., Barresi S., Barbalat R., Barton G.M., Granucci F., Kagan J.C. (2011). CD14 controls the LPS-induced endocytosis of Toll-like receptor 4. Cell.

[91] Pugin J., Schürer-Maly C.C., Leturcq D., Moriarty A., Ulevitch R.J., Tobias P.S. (1993). Lipopolysaccharide activation of human endothelial and epithelial cells is mediated by lipopolysaccharide-binding protein and soluble CD14. Proc. Natl. Acad. Sci. USA.

[92] Schulz C., Farkas L., Wolf K., Kratzel K., Eissner G., Pfeifer M. (2002). Differences in LPS-induced activation of bronchial epithelial cells (BEAS-2B) and type II-like pneumocytes (A-549). Scand. J. Immunol..

[93] Von Schéele I., Larsson K., Palmberg L. (2014). Interactions between alveolar epithelial cells and neutrophils under pro-inflammatory conditions. Eur. Clin. Respir. J..

[94] George C.L., White M.L., O’Neill M.E., Thorne P.S., Schwartz D.A., Snyder J.M. (2003). Altered surfactant protein A gene expression and protein metabolism associated with repeat exposure to inhaled endotoxin. Am. J. Physiol. Lung Cell. Mol. Physiol..

[95] Wang W.N., Zhou J.H., Wang P., Zhang X.J. (2016). The localization of SP-B and influences of lipopolysaccharide on it. Eur. Rev. Med. Pharmacol. Sci..

[96] Ingenito E.P., Mora R., Cullivan M., Marzan Y., Haley K., Mark L., Sonna L.A. (2001). Decreased surfactant protein-B expression and surfactant dysfunction in a murine model of acute lung injury. Am. J. Respir. Cell Mol. Biol..

[97] Lin J., Tian J., Wang L., Wu W., Li H., Wang X., Zeng X., Zhang W. (2017). Apoptosis and surfactant protein-C expression inhibition induced by lipopolysaccharide in AEC II cell may associate with NF-κB pathway. J. Toxicol. Sci..

[98] Kolomaznik M., Nova Z., Mokra D., Zila I., Kopincova J., Vidomanova E., Skovierova H., Halasova E., Calkovska A. (2018). Modified porcine surfactant restores lung homeostasis in LPS-challenged and artificially ventilated adult rats. Neonatology.

[99] Chuang C.Y., Chen T.L., Cherng Y.G., Tai Y.T., Chen T.G., Chen R.M. (2011). Lipopolysaccharide induces apoptotic insults to human alveolar epithelial A549 cells through reactive oxygen species-mediated activation of an intrinsic mitochondrion-dependent pathway. Arch. Toxicol..

[100] Kitamura Y., Hashimoto S., Mizuta N., Kobayashi A., Kooguchi K., Fujiwara I., Nakajima H. (2001). Fas/FasL-dependent apoptosis of alveolar cells after lipopolysaccharide-induced lung injury in mice. Am. J. Respir. Crit. Care Med..

[101] Vernooy J.H., Dentener M.A., van Suylen R.J., Buurman W.A., Wouters E.F. (2001). Intratracheal instillation of lipopolysaccharide in mice induces apoptosis in bronchial epithelial cells: No role for tumor necrosis factor-alpha and infiltrating neutrophils. Am. J. Respir. Cell Mol. Biol..

[102] Tang P.S., Mura M., Seth R., Liu M. (2008). Acute lung injury and cell death: How many ways can cells die?. Am. J. Physiol..

[103] Lin L., Zhang L., Yu L., Han L., Ji W., Shen H., Hu Z. (2016). Time-dependent changes of autophagy and apoptosis in lipopolysaccharide-induced rat acute lung injury. Iran. J. Basic Med. Sci..

[104] Kucukgul A., Eedogan S. (2017). Low concentration of oleic acid exacerbates LPS-induced cell death and inflammation in human alveolar epithelial cells. Exp. Lung Res..

[105] Chen R.M., Chen T.L., Lin Y.L., Chen T.G., Tai Y.T. (2005). Ketamine reduces nitric oxide biosynthesis in human umbilical vein endothelial cells by down-regulating endothelial nitric oxide synthase expression and intracellular calcium levels. Crit. Care Med..

[106] Lee C.J., Tai Y.T., Lin Y.L., Chen R.M. (2010). Molecular mechanisms of propofol-involved suppression of no biosynthesis and inducible iNOS gene expression in LPS-stimulated macrophage-like raw 264.7 cells. Shock.

[107] García-Verdugo I., Cañadas O., Taneva S.G., Keough K.M., Casals C. (2007). Surfactant protein A forms extensive lattice-like structures on 1,2-dipalmitoylphosphatidylcholine/rough-lipopolysaccharide-mixed monolayers. Biophys. J..

[108] Cañadas O., Keough K.M., Casals C. (2011). Bacterial lipopolysaccharide promotes destabilization of lung surfactant-like films. Biophys. J..

[109] Kishore U., Greenhough T.J., Waters P., Shrive A.K., Ghai R., Kamran M.F., Bernal A.L., Reid K.B.M., Madan T., Chakraborty T. (2006). Surfactant proteins SP-A and SP-D: Structure, function and receptors. Mol. Immunol..

[110] Gardai S.J., Xiao Y.Q., Dickinson M., Nick J.A., Voelker D.R., Greene K.E., Henson P.M. (2003). By binding SIRPalpha or calreticulin/CD91, lung collectins act as dual function surveillance molecules to suppress or enhance inflammation. Cell.

[111] Chaby R., Garcia-Verdugo I., Espinassous Q., Augusto L.A. (2005). Interactions between LPS and lung surfactant proteins. J. Endotoxin Res..

[112] Van Iwaarden J.F., Pikaar J.C., van Strijp J.A. (1994). Binding of surfactant protein A to the lipid A moiety of bacterial lipopolysaccharides. Biochem. J..

[113] Sano H., Chiba H., Iwaki D., Sohma H., Voelker D.R., Kuroki Y. (2000). Surfactant proteins A and D bind CD14 by different mechanisms. J. Biol. Chem..

[114] Wang H., Head J., Kosma P., Brade H., Müller-Loennies S., Sheikh S., McDonald B., Smith K., Cafarella T., Seaton B. (2008). Recognition of heptoses and the inner core of bacterial lipopolysaccharides by surfactant protein d. Biochemistry.

[115] Wu H., Kuzmenko A., Wan S., Schaffer L., Weiss A., Fisher J.H., Kim K.S., McCormack F.X. (2003). Surfactant proteins A and D inhibit the growth of Gram-negative bacteria by increasing membrane permeability. J. Clin. Investig..

[116] Cañadas O., García-Verdugo I., Keough K.M., Casals C. (2008). SP-A permeabilizes lipopolysaccharide membranes by forming protein aggregates that extract lipids from the membrane. Biophys. J..

[117] Keese S.P., Brandenburg K., Roessle M., Schromm A.B. (2014). Pulmonary surfactant protein A-induced changes in the molecular conformation of bacterial deep-rough LPS lead to reduced activity on human macrophages. Innate Immun..

[118] Chiba H., Sano H., Iwaki D., Murakami S., Mitsuzawa H., Takahashi T., Konishi M., Takahashi H., Kuroki Y. (2001). Rat mannose-binding protein a binds CD14. Infect. Immun..

[119] Murakami S., Iwaki D., Mitsuzawa H., Sano H., Takahashi H., Voelker D.R., Akino T., Kuroki Y. (2002). Surfactant protein A inhibits peptidoglycan-induced tumor necrosis factor-alpha secretion in U937 cells and alveolar macrophages by direct interaction with Toll-like receptor 2. J. Biol. Chem..

[120] Ohya M., Nishitani C., Sano H., Yamada C., Mitsuzawa H., Shimizu T., Saito T., Smith K., Crouch E., Kuroki Y. (2006). Human pulmonary surfactant protein D binds the extracellular domains of Toll-like receptors 2 and 4 through the carbohydrate recognition domain by a mechanism different from its binding to phosphatidylinositol and lipopolysaccharide. Biochemistry.

[121] Guillot L., Balloy V., McCormack F.X., Golenbock D.T., Chignard M., Si-Tahar M. (2002). Cutting edge: The immunostimulatory activity of the lung surfactant protein-A involves Toll-like receptor 4. J. Immunol..

[122] Yamada C., Sano H., Shimizu T., Mitsuzawa H., Nishitani C., Himi T., Kuroki Y. (2006). Surfactant Protein A Directly Interacts with TLR4 and MD-2 and Regulates Inflammatory Cellular Response. Importance of Supratrimeric Oligomerization. J. Biol. Chem..

[123] Mingarro I., Lukovic D., Vilar M., Pérez-Gil J. (2008). Synthetic pulmonary surfactant preparations: New developments and future trends. Curr. Med. Chem..

[124] Serrano A.G., Pérez-Gil J. (2006). Protein-lipid interactions and surface activity in the pulmonary surfactant system. Chem. Phys. Lipids.

[125] Augusto L.A., Li J., Synguelakis M., Johansson J., Chaby R. (2002). Structural basis for interactions between lung surfactant protein C and bacterial lipopolysaccharide. J. Biol. Chem..

[126] Augusto L.A., Synguelakis M., Espinassous Q., Lepoivre M., Johansson J., Chaby R. (2003). Cellular antiendotoxin activities of lung surfactant protein C in lipid vesicles. Am. J. Respir. Crit. Care Med..

[127] Glasser S.W., Senft A.P., Whitsett J.A., Maxfield M.D., Ross G.F., Richardson T.R., Prows D.R., Xu Y., Korfhagen T.R. (2008). Macrophage dysfunction and susceptibility to pulmonary Pseudomonas aeruginosa infection in surfactant protein C-deficient mice. J. Immunol..

[128] Augusto L.A., Synguelakis M., Johansson J., Pedron T., Girard R., Chaby R. (2003). Interaction of pulmonary surfactant protein C with CD14 and lipopolysaccharide. Infect. Immun..

[129] Cerutti C., Ridley A.J. (2017). Endothelial Cell-Cell Adhesion and Signaling. Exp. Cell Res..

[130] Aird W.C. (2008). Endothelium in health and disease. Pharmacol. Rep..

[131] Al-Soudi A., Kaaij M.H., Tas S.W. (2017). Endothelial Cells: From Innocent Bystanders to Active Participants in Immune Responses. Autoimmun. Rev..

[132] Mehta D., Malik A.B. (2006). Signaling mechanisms regulating endothelial permeability. Physiol. Rev..

[133] Janga H., Cassidy L., Wang F., Spengler D., Oestern-Fitschen S., Krause M.F., Seekamp A., Tholey A., Fuchs S. (2018). Site-Specific and Endothelial-Mediated Dysfunction of the Alveolar-Capillary Barrier in Response to Lipopolysaccharides. J. Cell. Mol. Med..

[134] Johnson E.R., Matthay M.A. (2010). Acute lung injury: Epidemiology, pathogenesis, and treatment. J. Aerosol Med. Pulm. Drug Deliv..

[135] Andonegui G., Sanna M. (2002). Goyert and Paul Kubes. Lipopolysaccharide-Induced Leukocyte-Endothelial Cell Interactions: A Role for CD14 Versus Toll-Like Receptor 4 Within Microvessels. J. Immunol..

[136] Bannerman D.D., Goldblum S.E. (2003). Mechanisms of bacterial lipopolysaccharide-induced endothelial apoptosis. Am. J. Physiol. Lung Cell. Mol. Physiol..

[137] Chao H.H., Chen P.Y., Hao W.R., Chiang W.P., Cheng T.H., Loh S.H., Leung Y.M., Liu J.C., Chen J.J., Sung L.C. (2017). Lipopolysaccharide pretreatment increases protease-activated receptor-2 expression and monocyte chemoattractant protein-1 secretion in vascular endothelial cells. J. Biomed. Sci..

[138] Aird W.C. (2003). The role of the endothelium in severe sepsis and multiple organ dysfunction syndrome. Blood.

[139] Petrache I., Birukova A., Ramirez S.I., Garcia J.G., Verin A.D. (2003). The role of the microtubules in tumor necrosis factor-alpha-induced endothelial cell permeability. Am. J. Respir. Cell Mol. Biol..

[140] Galkina E., Ley K. (2009). Immune and inflammatory mechanisms of atherosclerosis. Annu. Rev. Immunol..

[141] Sawa Y., Ueki T., Hata M., Iwasawa K., Tsuruga E., Kojima H., Ishikawa H., Yoshida S. (2008). LPS-induced IL-6, IL-8, VCAM-1, and ICAM-1 expression in human lymphatic endothelium. J. Histochem. Cytochem..

[142] Bolger M.S., Ross D.S., Jiang H., Frank M.M., Ghio A.J., Schwartz D.A., Wright J.R. (2007). Complement levels and activity in the normal and LPS-injured lung. Am. J. Physiol. Lung Cell. Mol. Physiol..

[143] Bosmann M., Ward P.A. (2012). Role of C3, C5 and anaphylatoxin receptors in acute lung injury and in sepsis. Adv. Exp. Med. Biol..

[144] Al-Sadi R., Ye D., Boivin M., Guo S., Hashimi M., Ereifej L., Ma T.Y. (2014). Interleukin-6 modulation of intestinal epithelial tight junction permeability is mediated by JNK pathway activation of claudin-2 gene. PLoS ONE.

[145] Reynier F., de Vos A.F., Hoogerwerf J.J., Bresser P., van der Zee J.S., Paye M., Pachot A., Mougin B., van der Poll T. (2012). Gene Expression Profiles in Alveolar Macrophages Induced by Lipopolysaccharide in Humans. Mol. Med..

[146] Forbes A., Pickell M., Foroughian M., Yao L.J., Lewis J., Veldhuizen R. (2007). Alveolar macrophage depletion is associated with increased surfactant pool sizes in adult rats. J. Appl. Physiol..

[147] Geissmann F., Manz M.G., Jung S., Sieweke M.H., Merad M., Ley K. (2010). Development of Monocytes, Macrophages, and Dendritic Cells. Science.

[148] Gordon S., Martinez F.O. (2010). Alternative Activation of Macrophages: Mechanism and Functions. Immunity.

[149] Hussell T., Bell T.J. (2014). Alveolar Macrophages: Plasticity in a Tissue-Specific Context. Nat. Rev. Immunol..

[150] Prasse A., Pechkovsky D.V., Toews G.B., Jungraithmayr W., Kollert F., Goldmann T., Vollmer E., Müller-Quernheim J., Zissel G. (2006). A Vicious Circle of Alveolar Macrophages and Fibroblasts Perpetuates Pulmonary Fibrosis via CCL18. Am. J. Respir. Crit. Care Med..

[151] Gibbons M.A., MacKinnon A.C., Ramachandran P., Dhaliwal K., Duffin R., Phythian-Adams A.T., van Rooijen N., Haslett C., Howie S.E., Simpson A.J. (2011). Ly6Chi Monocytes Direct Alternatively Activated Profibrotic Macrophage Regulation of Lung Fibrosis. Am. J. Respir. Crit. Care Med..

[152] Moreira A.P., Hogaboam C.M. (2011). Macrophages in Allergic Asthma: Fine-Tuning Their pro- and Anti-Inflammatory Actions for Disease Resolution. J. Interferon Cytokine Res..

[153] Tesfaigzi Y. (2006). Roles of Apoptosis in Airway Epithelia. Am. J. Respir. Cell Mol. Biol.

[154] Crosby L.M., Waters C.M. (2010). Epithelial Repair Mechanisms in the Lung. Am. J. Physiol. Lung Cell. Mol. Physiol..

[155] Herold S., Mayer K., Lohmeyer J. (2011). Acute Lung Injury: How Macrophages Orchestrate Resolution of Inflammation and Tissue Repair. Front. Immunol..

[156] Watford W.T., Wright J.R., Hester C.G., Jiang H., Frank M.M. (2001). Surfactant Protein A Regulates Complement Activation. J. Immunol..

[157] Henning L.N., Azad A.K., Parsa K.V., Crowther J.E., Tridandapani S., Schlesinger L.S. (2008). Pulmonary Surfactant Protein-A regulates Toll-like receptor expression and activity in human macrophages. J. Immunol..

[158] Arias-Diaz J., Garcia-Verdugo I., Casals C., Sanchez-Rico N., Vara E., Balibrea J.L. (2000). Effect of surfactant protein A (SP-A) on the production of cytokines by human pulmonary macrophages. Shock.

[159] Haczku A. (2008). Protective Role of the Lung Collectins Surfactant Protein A and Surfactant Protein D in Airway Inflammation. J. Allergy Clin. Immunol..

[160] Bufler P., Schmidt B., Schikor D., Bauernfeind A., Crouch E.C., Griese M. (2003). Surfactant protein A and D differently regulate the immune response to non-mucoid Pseudomonas aeruginosa and its lipopolysaccharide. Am. J. Respir. Cell Mol. Biol..

[161] Snelgrove R.J., Goulding J., Didierlaurent A.M., Lyonga D., Vekaria S., Edwards L., Gwyer E., Sedgwick J.D., Barclay A.N., Hussell T. (2008). A Critical Function for CD200 in Lung Immune Homeostasis and the Severity of Influenza Infection. Nat. Immunol..

[162] Imai Y., Kuba K., Neely G.G., Yaghubian-Malhami R., Perkmann T., van Loo G., Ermolaeva M., Veldhuizen R., Leung Y.H., Wang H. (2008). Identification of oxidative stress and Toll-like receptor 4 signaling as a key pathway of acute lung injury. Cell.

[163] Adib-Conquy M., Moine P., Asehnoune K., Edouard A., Espevik T., Miyake K., Werts C., Cavaillon J.M. (2003). Toll-like Receptor-mediated Tumor Necrosis Factor and Interleukin-10 Production Differ during Systemic Inflammation. Am. J. Respir. Crit. Care Med..

[164] Chen L.C., Gordon R.E., Laskin J.D., Laskin D.L. (2007). Role of TLR-4 in Liver Macrophage and Endothelial Cell Responsiveness During Acute Endotoxemia. Exp. Mol. Pathol..

[165] Hoogerwerf J.J., de Vos A.F., Bresser P., van der Zee J.S., Pater J.M., de Boer A., Tanck M., Lundell D.L., Her-Jenh C., Draing C. (2008). Lung Inflammation Induced by Lipoteichoic Acid or Lipopolysaccharide in Humans. Am. J. Respir. Crit. Care Med..

[166] Islam M.A., Pröll M., Hölker M., Tholen E., Tesfaye D., Looft C., Schellander K., Cinar M.U. (2013). Alveolar Macrophage Phagocytic Activity Is Enhanced with LPS Priming, and Combined Stimulation of LPS and Lipoteichoic Acid Synergistically Induce pro-Inflammatory Cytokines in Pigs. Innate Immun..

[167] Islam M.A., Cinar M.U., Uddin M.J., Tholen E., Tesfaye D., Looft C., Schellander K. (2012). Expression of Toll-like receptors and downstream genes in lipopolysaccharide-induced porcine alveolar macrophages. Vet. Immunol. Immunopathol..

[168] Feng X., Deng T., Zhang Y., Su S., Wei C., Han D. (2011). Lipopolysaccharide inhibits macrophage phagocytosis of apoptotic neutrophils by regulating the production of tumour necrosis factor alpha and growth arrest-specific gene 6. Immunology.

[169] Vaccaro C., Brody J.S. (1978). Ultrastructure of developing alveoli. I. The role of the interstitial fibroblast. Anat. Rec..

[170] Ruiz-Camp J., Morty R.E. (2015). Divergent fibroblast growth factor signaling pathways in lung fibroblast subsets: Where do we go from here?. Am. J. Physiol. Lung Cell. Mol. Physiol..

[171] Hinz B., Phan S.H., Thannickal V.J., Galli A., Bochaton-Piallat M.L., Gabbiani G. (2007). The myofibroblast: one function, multiple origins. Am. J. Pathol..

[172] He Z., Zhu Y., Jiang H. (2009). Toll-like receptor 4 mediates lipopolysaccharide-induced collagen secretion by phosphoinositide3-kinase-Akt pathway in fibroblasts during acute lung injury. J. Recept. Signal Transduct. Res..

[173] He Z., Gao Y., Deng Y., Li W., Chen Y., Xing S., Zhao X., Ding J., Wang X. (2012). Lipopolysaccharide induces lung fibroblast proliferation through Toll-like receptor 4 signaling and the phosphoinositide3-kinase-Akt pathway. PLoS ONE.

[174] Chen X., Sun R., Hu J., Mo Z., Yang Z., Liao D., Zhong N. (2008). Attenuation of bleomycin-induced lung fibrosis by oxymatrine is associated with regulation of fibroblast proliferation and collagen production in primary culture. Basic Clin. Pharmacol. Toxicol..

[175] Xia H., Diebold D., Nho R., Perlman D., Kleidon J., Kahm J., Avdulov S., Peterson M., Nerva J., Bitterman P. (2008). Pathological integrin signaling enhances proliferation of primary lung fibroblasts from patients with idiopathic pulmonary fibrosis. J. Exp. Med..

[176] He Z., Zhu Y., Jiang H. (2009). Inhibiting toll-like receptor 4 signaling ameliorates pulmonary fibrosis during acute lung injury induced by lipopolysaccharide: An experimental study. Respir. Res..

[177] Sakai N., Tager A.M. (2013). Fibrosis of two: Epithelial cell-fibroblast interactions in pulmonary fibrosis. Biochim. Biophys. Acta.

